# Inequalities for the Casorati Curvature of Totally Real Spacelike Submanifolds in Statistical Manifolds of Type Para-Kähler Space Forms

**DOI:** 10.3390/e23111399

**Published:** 2021-10-25

**Authors:** Bang-Yen Chen, Simona Decu, Gabriel-Eduard Vîlcu

**Affiliations:** 1 Department of Mathematics, Michigan State University, East Lansing, MI 48824-1027, USA; chenb@msu.edu; 2 Department of Applied Mathematics, Bucharest University of Economic Studies, 6 Piaţa Romană, 010374 Bucharest, Romania; 3 Romanian Academy, *Costin C. Kiriţescu* National Institute of Economic Research, Centre of Mountain Economy (CE-MONT), 13 Calea 13 Septembrie, 030508 Bucharest, Romania; 4 Faculty of Mathematics and Computer Science, Research Center in Geometry, Topology and Algebra, University of Bucharest, Str. Academiei 14, Sector 1, 70109 Bucharest, Romania; gvilcu@upg-ploiesti.ro; 5 Department of Cybernetics, Economic Informatics, Finance and Accountancy, Petroleum-Gas University of Ploieşti, Bd. Bucureşti 39, 100680 Ploieşti, Romania

**Keywords:** Casorati curvature, statistical manifold of type para-Kähler space form, totally real submanifold

## Abstract

The purpose of this article is to establish some inequalities concerning the normalized δ-Casorati curvatures (extrinsic invariants) and the scalar curvature (intrinsic invariant) of totally real spacelike submanifolds in statistical manifolds of the type para-Kähler space form. Moreover, this study is focused on the equality cases in these inequalities. Some examples are also provided.

## 1. Introduction

One of the basic problems in information geometry is to study the geometric properties of a *statistical manifold*, a concept introduced by Amari [1]. There is great interest in researching statistical manifolds with applications not only in information geometry but also in differential geometry, physics, statistics, machine learning, etc. The natural relationship between statistical manifolds and *entropy* has been investigated by many researchers. Mishra and Kumar studied in [2] the structure of statistical manifolds with respect to a relative α-entropy in a Bayesian framework to obtain a generalized Bayesian Cramér-Rao inequality. The entropic dynamics on statistical manifolds of Gibbs distributions are investigated in [3], which can provide new insight in fields such as economics and ecology. Very recently, the authors in [4] studied the information-geometric properties of the statistical manifold to reduce predictive uncertainly via data assimilation. On the other hand, statistical manifolds provide a setting for the theory of submanifolds, where a basic problem is to find out simple relationships between the main intrinsic and extrinsic curvature invariants of submanifolds [5]. In this regard, many geometers studied certain types of geometric inequalities in (statistical) submanifolds. Comprehensive surveys on such inequalities are provided by Chen in [5,6,7].

Recently, there has been growing interest in the study of optimal inequalities involving the extrinsic *δ-Casorati curvatures* defined by Decu, Haesen, and Verstraelen in [8,9]. A long time ago, Casorati introduced a new measure of curvature of a surface (now called the *Casorati curvature*) following a *common idea of curvature*, more accurate than the Gauss and mean curvature [10]. Only in modern times did mathematical models involving Casorati curvature begin to be studied, e.g., in computer vision [11]. Furthermore, Verstraelen described qualitatively geometrical models for human early vision [12]. In this respect, the corresponding (visual) perceptions can be defined as the surfaces $M^{2}$ given by the Casorati curvatures $C=\frac{1}{2}\left(k_{1}^{2}+k_{2}^{2}\right)$ in $E^{3}$, where $k_{1}$ and $k_{2}$ are the principal curvatures of $M^{2}$ in $E^{3}$ [12]. A geometrical interpretation of this curvature for submanifolds in Riemannian spaces was proved in [13]. In economics, the isotropical Casorati curvature of production surfaces was investigated in [14]. Initially, the δ-Casorati curvatures were studied in optimal inequalities for submanifolds in real space forms in [8,9]. Later, this topic was extensively studied (e.g., see [5]). Recently, Suceavă and Vajiac established inequalities involving some Chen invariants, mean curvature, and Casorati curvature for strictly convex Euclidean hypersurfaces [15].

*Para-Kähler geometry* refers to the algebra of para-complex numbers (or hyperbolic numbers) and, especially, to the study of para-Kähler structures and their derived forms. Para-complex numbers were defined by Graves [16] in 1845 as a generalization of complex numbers using the expression x+yj, where *x* and *y* ∈R, and *j* satisfies $j^{2}=1$ and j≠1. *Para-Kähler manifolds* were first investigated as *stratified spaces* by Rashevskij [17] (1948). These manifolds were explicitly defined independently in 1949 by Rozenfeld [18] and Ruse [19]. This is a challenging topic now, related to many applications in mathematics, physics, and mechanics [20]. Defever, Deszcz, and Verstraelen considered para-Kähler manifolds that satisfy curvature conditions of the pseudosymmetric type, with applications in the theory of general relativity [21]. Mihai and Rosca dealt with CR-submanifolds of para-Kählerian manifolds, which carry skew-symmetric vector fields [22]. Recently, Fei and Zhang introduced in [23] the notion of a Codazzi-para-Kähler structure and studied the interaction of Codazzi couplings with para-Kähler geometry; essentially, a Codazzi-para-Kähler structure is simultaneously a statistical structure and a para-Kähler structure [23]. Very recently, Vîlcu investigated statistical manifolds endowed with almost product structures and para-Kähler-like statistical submersions [24].

*Totally real* and, particularly, *Lagrangian submanifolds* in Kähler manifolds, complex space forms, etc. have been explored widely (see, for instance, [25,26,27,28,29,30,31]). However, not much is known about totally real and Lagrangian submanifolds in para-Kähler manifolds. Chen proved general optimal inequalities involving the scalar curvature and mean curvature for Lagrangian submanifolds of the flat para-Kähler manifold $\left(E^{2n},g,P\right)$ [32]. In addition, he studied Lagrangian *H*-umbilical submanifolds of para-Kähler manifolds [33]. Anciaux and Georgiou surveyed the Hamilton stability of Hamiltonian minimal Lagrangian submanifolds in para-Kähler manifolds [34].

In this paper, we establish optimal inequalities between the intrinsic scalar curvature and the extrinsic δ-Casorati curvatures of totally real spacelike submanifolds of statistical manifolds of the type para-Kähler space form. Moreover, we investigate the equality cases in these inequalities. We present also some examples.

## 2. Preliminaries

Let ($\bar{M}$, $\bar{g}$) be a semi-Riemannian manifold, let $\bar{g}$ be a semi-Riemannian metric on $\bar{M}$, and let $\tilde{\nabla }$ be an affine connection on $\tilde{M}$.

In the following, we will denote by ${\bar{\nabla }}^{*}$ the *conjugate (dual) affine connection* of $\bar{\nabla }$, expressed by

$$X_{1}\;\tilde{g}\left(X_{2},X_{3}\right)=\bar{g}\left({\bar{\nabla }}_{X_{1}}X_{2},X_{3}\right)+\bar{g}\left(X_{2},{\bar{\nabla }}_{X_{1}}^{*}X_{3}\right),$$

for any $X_{1}$, $X_{2}$, $X_{3}$ $\in \Gamma (T\bar{M})$, where $\Gamma (T\bar{M})$ is the set of smooth tangent vector fields on $\bar{M}$.

If the torsion tensor field of $\tilde{\nabla }$ vanishes and $\bar{\nabla }\bar{g}$ is symmetric, then $\left(\bar{\nabla },\bar{g}\right)$ is called a *statistical structure* on $\bar{M}$. Thus, ($\bar{M}$, $\bar{g}$, $\bar{\nabla }$) is named a *statistical manifold* [35]. Clearly, if $(\bar{M}$, $\bar{g}$, $\bar{\nabla })$ is a statistical manifold, then $(\bar{M}$, $\bar{g}$, ${\bar{\nabla }}^{*})$ is as well. Moreover, one easily can see that $\bar{\nabla }={\left({\bar{\nabla }}^{*}\right)}^{*}$ and ${\bar{\nabla }}^{0}=\frac{\bar{\nabla }+{\bar{\nabla }}^{*}}{2}$, where ${\bar{\nabla }}^{0}$ is the Levi–Civita connection of $\bar{M}$ [36].

Let *N* be an *n*-dimensional submanifold of a 2m-dimensional statistical manifold $(\bar{M}$, $\bar{g}$, $\bar{\nabla })$, with *g* as the induced metric on *N* and ∇ as the induced connection on *N*. (N,g,∇) is then also a statistical manifold. For any $X_{1},X_{2}\in \Gamma \left(TN\right)$, we have the following *formulas of Gauss* [35]:

$$\begin{array}{c} {\tilde{\nabla }}_{X_{1}}X_{2}=\nabla_{X_{1}}X_{2}+h\left(X_{1},X_{2}\right),\\ {\tilde{\nabla }}_{X_{1}}^{*}X_{2}=\nabla_{X_{1}}^{*}X_{2}+h^{*}\left(X_{1},X_{2}\right), \end{array}$$

where the (0,2)-tensors *h* and $h^{*}$ (bilinear and symmetric) are said to be the *imbedding curvature tensor* of *N* in $\bar{M}$ with respect to $\bar{\nabla }$ and ${\bar{\nabla }}^{*}$, respectively.

The *mean curvature* vector fields of *N* for $\bar{\nabla }$ and ${\bar{\nabla }}^{*}$ are given by, respectively,

$$H=\frac{1}{n}tr_{g}\left(h\right),\;\;H^{*}=\frac{1}{n}tr_{g}\left(h^{*}\right),$$

where $tr_{g}$ is the trace with respect to *g*.

For the Levi–Civita connection ${\bar{\nabla }}^{0}$, we denote by $h^{0}=\frac{h+h^{*}}{2}$ the *second fundamental form* and by

(1) $$H^{0}=\frac{H+H^{*}}{2}$$

the *mean curvature* vector field of *N*.

Let $\left\{e_{1},\ldots ,e_{n}\right\}$ and $\left\{e_{n+1},\ldots ,e_{2m}\right\}$ be orthonormal bases of the tangent space $T_{x}N$ and $T_{x}^{⊥}N$, respectively, at a point x∈N. The *squared mean curvatures* of *N* for ∇ and $\nabla^{*}$ then satisfy

$${∥H∥}^{2}=\frac{1}{n^{2}}\sum_{\alpha =n+1}^{2m}{\left(\sum_{i=1}^{n}h_{ii}^{\alpha }\right)}^{2},\;\;{∥H^{*}∥}^{2}=\frac{1}{n^{2}}\sum_{\alpha =n+1}^{2m}{\left(\sum_{i=1}^{n}h_{ii}^{*\alpha }\right)}^{2},$$

where $h_{ij}^{\alpha }=g\left(h\left(e_{i},e_{j}\right),e_{\alpha }\right)$ and $h_{ij}^{*\alpha }=g\left(h^{*}\left(e_{i},e_{j}\right),e_{\alpha }\right)$ for i,j∈{1,…,n}, α∈{n+1,…,2m}.

Denote by C and $C^{*}$ the *Casorati curvatures* of the submanifold *N*, defined by the squared norms of *h* and $h^{*}$, respectively, over the dimension *n* of *N*, as follows:

$$C=\frac{1}{n}{∥h∥}^{2}=\frac{1}{n}\sum_{\alpha =n+1}^{2m}\sum_{i,j=1}^{n}{\left(h_{ij}^{\alpha }\right)}^{2},$$

$$C^{*}=\frac{1}{n}{∥h^{*}∥}^{2}=\frac{1}{n}\sum_{\alpha =n+1}^{2m}\sum_{i,j=1}^{n}{\left(h_{ij}^{*\alpha }\right)}^{2}.$$

We denote by $C^{0}$ the following expression:

(2) $$C^{0}=\frac{C+C^{*}}{2}.$$

Consider *V* as an *k*-dimensional subspace of $T_{x}N$, k≥2, and $\left\{e_{1},\ldots ,e_{k}\right\}$ as an orthonormal frame of *V*. Thus, the Casorati curvatures C(V) and $C^{*}\left(V\right)$ of *V* are revealed by

$$C\left(V\right)=\frac{1}{k}\sum_{\alpha =n+1}^{2m}\sum_{i,j=1}^{k}{\left(h_{ij}^{\alpha }\right)}^{2},\;\;C^{*}\left(V\right)=\frac{1}{k}\sum_{\alpha =n+1}^{2m}\sum_{i,j=1}^{k}{\left(h_{ij}^{*\alpha }\right)}^{2}.$$

Denote by $\delta_{C}\left(r;n-1\right)$ and ${\hat{\delta }}_{C}\left(r;n-1\right)$ the *generalized normalized δ-Casorati curvatures* of *N*, defined in [9] as

$$\delta_{C}{\left(r;n-1\right)|}_{x}=r\;C{|}_{x}+a\left(r\right)\;\inf\left\{C\left(V\right)|V\;a\;\mathrm{hyperplane}\;\mathrm{of}\;T_{x}N\right\},$$

if 0<r<n(n−1), and

$${\hat{\delta }}_{C}{\left(r;n-1\right)|}_{x}=r\;C{|}_{x}+a\left(r\right)\;\sup\left\{C\left(V\right)|V\;a\;\mathrm{hyperplane}\;\mathrm{of}\;T_{x}N\right\},$$

if r>n(n−1), for an a(r) set as

$$\begin{array}{c} a\left(r\right)=\frac{\left(n-1\right)\left(r+n\right)\left(n^{2}-n-r\right)}{nr}, \end{array}$$

where $r\in R_{+}$ and r≠n(n−1).

Additionally, denote by $\delta_{C}^{*}\left(r;n-1\right)$ and ${\hat{\delta^{*}}}_{C}\left(r;n-1\right)$ the dual generalized normalized $\delta^{*}$-Casorati curvatures of the submanifold *N*, defined as follows:

$$\delta_{C}^{*}{\left(r;n-1\right)|}_{x}=r\;C^{*}{|}_{x}+a\left(r\right)\;\inf\left\{C^{*}\left(V\right)|V\;a\;\mathrm{hyperplane}\;\mathrm{of}\;T_{x}N\right\},$$

if 0<r<n(n−1), and

$${\hat{\delta }}_{C}^{*}{\left(r;n-1\right)|}_{x}=r\;C^{*}{|}_{x}+a\left(r\right)\;\sup\left\{C^{*}\left(V\right)|V\;a\;\mathrm{hyperplane}\;\mathrm{of}\;T_{x}N\right\},$$

if r>n(n−1), for the a(r) set above.

The *normalized δ-Casorati curvatures* $\delta_{C}\left(n-1\right)$ and ${\hat{\delta }}_{C}\left(n-1\right)$ of the submanifold *N* are defined by

$$\delta_{C}{\left(n-1\right)|}_{x}=\frac{1}{2}C{|}_{x}+\frac{n+1}{2n}\inf\left\{C\left(V\right)|V\;a\;\mathrm{hyperplane}\;\mathrm{of}\;T_{x}N\right\}$$

and

$${\hat{\delta }}_{C}{\left(n-1\right)|}_{x}=2C{|}_{x}-\frac{2n-1}{2n}\sup\left\{C\left(V\right)|V\;a\;\mathrm{hyperplane}\;\mathrm{of}\;T_{x}N\right\}.$$

Furthermore, the dual normalized $\delta^{*}$-Casorati curvatures $\delta_{C}^{*}\left(n-1\right)$ and ${\hat{\delta }}_{C}^{*}\left(n-1\right)$ of the submanifold *N* in $\bar{M}$ can be written as

$$\delta_{C}^{*}{\left(n-1\right)|}_{x}=\frac{1}{2}C^{*}{|}_{x}+\frac{n+1}{2n}\inf\left\{C^{*}\left(V\right)|V\;a\;\mathrm{hyperplane}\;\mathrm{of}\;T_{x}N\right\}$$

and

$${\hat{\delta }}_{C}^{*}{\left(n-1\right)|}_{x}=2C^{*}{|}_{x}-\frac{2n-1}{2n}\sup\left\{C^{*}\left(V\right)|V\;a\;\mathrm{hyperplane}\;\mathrm{of}\;T_{x}N\right\}.$$

A statistical submanifold (N,g,∇) of $\left(\bar{M},\bar{g},\bar{\nabla }\right)$ is said to be *totally geodesic* with respect to $\bar{\nabla }$ if the second fundamental form of *N* vanishes identically [35].

Let *R* and $\bar{R}$ be the (0,4)-*curvature tensors* for the connections ∇ and $\bar{\nabla }$, respectively.

For the vector fields $X_{1},X_{2},X_{3},X_{4}$ tangent to *N*, the *equation of Gauss* on the connection $\bar{\nabla }$ is then [36]

(3) $$\begin{array}{ccc} \bar{g}\left(\bar{R}\left(X_{1},X_{2}\right)X_{3},X_{4}\right) & = & g\left(R\left(X_{1},X_{2}\right)X_{3},X_{4}\right)+\bar{g}\left(h\left(X_{1},X_{3}\right),h^{*}\left(X_{2},X_{4}\right)\right)\\ & - & \bar{g}\left(h^{*}\left(X_{1},X_{4}\right),h\left(X_{2},X_{3}\right)\right). \end{array}$$

Similarly, let $R^{*}$ and ${\bar{R}}^{*}$ be the (0,4)-*curvature tensors* for the connections $\nabla^{*}$ and ${\bar{\nabla }}^{*}$, respectively.

Next, for the vector fields $X_{1},X_{2},X_{3},X_{4}$ tangent to *N*, the *equation of Gauss* on the connection ${\bar{\nabla }}^{*}$ becomes [36]

(4) $$\begin{array}{ccc} \bar{g}\left({\bar{R}}^{*}\left(X_{1},X_{2}\right)X_{3},X_{4}\right) & = & g\left(R^{*}\left(X_{1},X_{2}\right)X_{3},X_{4}\right)+\bar{g}\left(h^{*}\left(X_{1},X_{3}\right),h\left(X_{2},X_{4}\right)\right)\\ & - & \bar{g}\left(h\left(X_{1},X_{4}\right),h^{*}\left(X_{2},X_{3}\right)\right). \end{array}$$

In general, $g(R\left(X_{1},X_{2}\right)X_{3},X_{4})$ is not skew-symmetric for $X_{3}$, $X_{4}$ [37], i.e.,

(5) $$\begin{array}{c} g\left(R\left(X_{1},X_{2}\right)X_{3},X_{4}\right)\neq -g\left(R\left(X_{1},X_{2}\right)X_{4},X_{3}\right). \end{array}$$

In order to define the sectional curvature of a statistical manifold, the property (5) of *R* is inconvenient. To overcome this, we define according to [35] the *statistical curvature tensor field* denoted by *S* for the statistical manifold (N,g,∇):

(6) $$S\left(X_{1},X_{2}\right)X_{3}=\frac{1}{2}\left\{R\left(X_{1},X_{2}\right)X_{3}+R^{*}\left(X_{1},X_{2}\right)X_{3}\right\},$$

for $X_{1},X_{2},X_{3}\in \Gamma \left(TN\right)$. Naturally, *S* is skew-symmetric relative to $X_{3}$, $X_{4}$. Moreover, *S* satisfies the first Bianchi identity. It follows that *S* is a Riemann-curvature-like tensor [37].

For a non-degenerate 2-dimensional subspace π of the tangent space $T_{x}N$, at a point x∈N, we can define immediately the *sectional curvature* of (N,∇,g) [35] by

(7) $$\begin{array}{c} K\left(\pi \right)=K\left(X\wedge Y\right)=\frac{g(S(X,Y)Y,X)}{g\left(X,X\right)g\left(Y,Y\right)-g^{2}\left(X,Y\right)}, \end{array}$$

where {X,Y} is a basis of π.

The *scalar curvature* τ of (N,∇,g) at a point x∈N is defined by the following expression:

(8) $$\begin{array}{c} \tau \left(x\right)=\sum_{1\leq i<j\leq n}K\left(e_{i}\wedge e_{j}\right)=\sum_{1\leq i<j\leq n}g\left(S\left(e_{i},e_{j}\right)e_{j},e_{i}\right), \end{array}$$

where $\left\{e_{1},\ldots ,e_{n}\right\}$ is an orthonormal frame at *x*.

Moreover, the *normalized scalar curvature* ρ of *N* is defined as

(9) $$\rho =\frac{2\tau }{n(n-1)}.$$

An *almost product structure* on a smooth manifold $\bar{M}$ is a (1,1)-tensor field P≠±I, such that

$$P^{2}=I,$$

where *I* is the identity tensor field of type (1,1) on $\bar{M}$. A manifold $\bar{M}$ is called an *almost para-Hermitian manifold* [7] if $\bar{M}$ is endowed with an almost product structure *P* and a semi-Riemannian metric $\bar{g}$ such that

(10) $$\begin{array}{c} \bar{g}\left(PX_{1},PX_{2}\right)=-\bar{g}\left(X_{1},X_{2}\right), \end{array}$$

for all vector fields $X_{1}$, $X_{2}$ on $\bar{M}$.

Hence, the dimension of an almost para-Hermitian manifold denoted by $\left(\bar{M},P,\bar{g}\right)$ is even, i.e., dim $\bar{M}=2m$, and the metric is neutral. If $\bar{\nabla }P=0$, then $\left(\bar{M},P,\bar{g}\right)$ is named a *para-Kähler manifold* [7], where $\bar{\nabla }$ is the Levi–Civita connection of $\bar{M}$.

Next, a triple $\left(\bar{M},P,\bar{g}\right)$ is said to be an *almost para-Hermitian-like manifold* [24] if a semi-Riemannian manifold $\left(\bar{M},\bar{g}\right)$ is endowed with an almost product structure *P* such that

(11) $$\bar{g}\left(PX_{1},X_{2}\right)+\bar{g}\left(X_{1},P^{*}X_{2}\right)=0,$$

for all vector fields $X_{1}$, $X_{2}$ on $\bar{M}$, where $P^{*}$ is (1,1)-tensor field on $\bar{M}$.

Let $\left(\bar{M},P,\bar{g}\right)$ be an almost para-Hermitian-like manifold. If $\left(\bar{\nabla },\bar{g}\right)$ is a statistical structure on $\bar{M}$ such that $\bar{\nabla }P=0$, then $\left(\bar{M},\bar{\nabla },P,\bar{g}\right)$ is called a *para-Kähler-like statistical manifold* [24].

Consequently, the notion of a para-Kähler-like statistical manifold is a generalization of the notion of the para-Kähler manifold (when, in particular, we have $P=P^{*}$, i.e., Formula (11) reduces to Formula (10), and $\bar{\nabla }$ is the Levi–Civita connection).

A para-Kähler-like statistical manifold $\left(\bar{M},\bar{\nabla },P,\bar{g}\right)$ is called a *statistical manifold of a type para-Kähler space form* if the following formula holds [24]:

(12) $$\begin{array}{c} \bar{R}\left(X_{1},X_{2}\right)X_{3}=\frac{c}{4}\{\bar{g}\left(X_{2},X_{3}\right)X_{1}-\bar{g}\left(X_{1},X_{3}\right)X_{2}+\bar{g}\left(PX_{2},X_{3}\right)PX_{1}\\ -\bar{g}\left(PX_{1},X_{3}\right)PX_{2}+\bar{g}\left(X_{1},PX_{2}\right)PX_{3}-\bar{g}\left(PX_{1},X_{2}\right)PX_{3}\}, \end{array}$$

for any vector fields $X_{1},X_{2},X_{3}$, where $\bar{R}$ is the curvature tensor of the connection $\bar{\nabla }$, and *c* is a real constant.

A submanifold *N* in an almost para-Hermitian (like) manifold $\left(\bar{M},P,\bar{g}\right)$ is called *totally real* if *P* maps each tangent space $T_{x}N$ into its corresponding normal space $T_{x}^{⊥}N$. In the particular case, when *P* interchanges each tangent space with its corresponding normal space, then *N* is said to be *Lagrangian*.

Next, we consider the constrained extremum problem

(13) $$\min_{x\in N}f\left(x\right),$$

where *N* is a submanifold of a (semi)-Riemannian manifold $\left(\bar{M},\bar{g}\right)$, and $f:\bar{M}\to R$ is a function of differentiability class $C^{2}$.

**Theorem** **1** ([38])**.** *If N is complete and connected, $\left(gradf\right)\left(x_{0}\right)\in T_{x_{0}}^{⊥}N$ for a point $x_{0}\in N$, and the bilinear form $F:T_{x_{0}}N\times T_{x_{0}}N\to R$ defined by*

(14) $$F\left(X_{1},X_{2}\right)=\mathrm{Hess}\left(f\right)\left(X_{1},X_{2}\right)+\bar{g}\left(h\left(X_{1},X_{2}\right),\mathrm{grad}f\right)$$

*is positive definite in $x_{0}$, then $x_{0}$ is the optimal solution of the problem *(13)*, where h is the second fundamental form of N.*

**Remark** **1** ([38])**.** *If the bilinear form F defined by *(14)* is positive semi-definite on the submanifold N, then the critical points of f|N are global optimal solutions of the problem *(13)*.*

## 3. Main Inequalities

It is well-known that one of the most fundamental problems in submanifold theory is the following (see, e.g., [39]).

Problem. *Establish a simple relationship between the main extrinsic invariants and the main intrinsic invariants of a submanifold.*

The following theorem provides an answer to this problem.

**Theorem** **2.** *Let N be an n-dimensional totally real spacelike submanifold of a 2m-dimensional statistical manifold of a type para-Kähler space form ($\bar{M},\bar{\nabla },P,\bar{g}$). The following inequalities involving the generalized normalized δ-Casorati curvatures then hold:*

*(i)* 
(15) $$\begin{array}{ccc} \delta_{C}^{0}\left(r;n-1\right) & \geq & 2\tau +2n^{2}{∥H^{0}∥}^{2}-nC^{0}-n^{2}\bar{g}\left(H,H^{*}\right)+\frac{c}{4}n\left(1-n\right), \end{array}$$
  where r∈R such that 0<r<n(n−1), $2\delta_{C}^{0}\left(r;n-1\right)=\delta_{C}\left(r;n-1\right)+\delta_{C}^{*}\left(r;n-1\right)$, $2C^{0}=C+C^{*}$, and
*(ii)* 
(16) $$\begin{array}{ccc} {\hat{\delta }}_{C}^{0}\left(r;n-1\right) & \geq & 2\tau +2n^{2}{∥H^{0}∥}^{2}-nC^{0}-n^{2}\bar{g}\left(H,H^{*}\right)+\frac{c}{4}n\left(1-n\right), \end{array}$$
  where r∈R such that r>n(n−1), $2{\hat{\delta }}_{C}^{0}\left(r;n-1\right)={\hat{\delta }}_{C}\left(r;n-1\right)+{\hat{\delta }}_{C}^{*}\left(r;n-1\right)$.

*Moreover, the equality sign of *(15)* and *(16)* holds identically at all points x∈N if and only if we have*

(17) $$h+h^{*}=0,$$

where h and $h^{*}$ are the imbedding curvature tensors of the submanifold associated to the dual connections $\tilde{\nabla }$ and ${\tilde{\nabla }}^{*}$, respectively.

**Proof.**  From Formulas (3), (4), and (6), it follows that

(18) $$\begin{array}{c} 2\bar{g}\left(\bar{S}\left(X_{1},X_{2}\right)X_{3},X_{4}\right)=2g\left(S\left(X_{1},X_{2}\right)X_{3},X_{4}\right)-\bar{g}\left(h\left(X_{2},X_{3}\right),h^{*}\left(X_{1},X_{4}\right)\right)\\ +\bar{g}\left(h\left(X_{1},X_{3}\right),h^{*}\left(X_{2},X_{4}\right)\right)-\bar{g}\left(h^{*}\left(X_{2},X_{3}\right),h\left(X_{1},X_{4}\right)\right)+\bar{g}\left(h^{*}\left(X_{1},X_{3}\right),h\left(X_{2},X_{4}\right)\right), \end{array}$$

where $X_{1},X_{2},X_{3},X_{4}\in \Gamma \left(TN\right)$.

For x∈N, let $\left\{e_{1},\ldots ,e_{n}\right\}$ be an orthonormal frame in $T_{x}N$, and let $\left\{e_{n+1},\ldots ,e_{2m}\right\}$ be an orthonormal frame in $T_{x}^{⊥}N$. Considering $X_{1}=X_{3}=e_{i}$ and $X_{2}=X_{4}=e_{j}$ with i,j∈{1,…,n}, from Equation (18), we obtain

(19) $$\begin{array}{ccc} 2\tau (x) & = & n^{2}\bar{g}\left(H,H^{*}\right)-\sum_{1\leq i,j\leq n}\bar{g}\left(h\left(e_{i},e_{j}\right),h^{*}\left(e_{i},e_{j}\right)\right)+\frac{c}{4}n\left(n-1\right). \end{array}$$

Using notations $H^{0}$ and $C^{0}$ given by (1) and (2), then Equation (19) can be written as

(20) $$\begin{array}{ccc} 2\tau (x) & = & \frac{c}{4}n\left(n-1\right)+2n^{2}∥H^{0}{∥}^{2}-\frac{n^{2}}{2}{∥H∥}^{2}-\frac{n^{2}}{2}{∥H^{*}∥}^{2}\\ & & -2nC^{0}+\frac{n}{2}\left(C+C^{*}\right). \end{array}$$

We choose Q, a quadratic polynomial expressed by

(21) $$\begin{array}{ccc} Q & = & rC^{0}+a\left(r\right)\;C^{0}\left(V\right)+\frac{n}{2}\left(C+C^{*}\right)-\frac{n^{2}}{2}{(∥H∥}^{2}+∥H^{*}{∥}^{2})\\ & & -2\tau \left(x\right)+\frac{c}{4}n\left(n-1\right), \end{array}$$

where *V* is a hyperplane of $T_{x}N$.

Let *V* be a hyperplane spanned by the tangent vectors $e_{1},\ldots ,e_{n-1}$, without losing the generality. We see that

(22) $$\begin{array}{c} Q=\sum_{\alpha =n+1}^{2m}\left[\frac{2n+r}{n}\sum_{i,j=1}^{n}{\left(h_{ij}^{0\alpha }\right)}^{2}+a\left(r\right)\frac{1}{n-1}\sum_{i,j=1}^{n-1}{\left(h_{ij}^{0\alpha }\right)}^{2}-2{\left(\sum_{i=1}^{n}h_{ii}^{0\alpha }\right)}^{2}\right]. \end{array}$$

Moreover, (22) becomes

$$\begin{array}{ccc} Q & = & \sum_{\alpha =n+1}^{2m}\{\left[\frac{2(2n+r)}{n}+\frac{2a(r)}{n-1}\right]\sum_{1\leq i<j\leq n-1}{\left(h_{ij}^{0\alpha }\right)}^{2}+\left[\frac{2(2n+r)}{n}+\frac{2a(r)}{n-1}\right]\sum_{i=1}^{n-1}{\left(h_{i\;n}^{0\alpha }\right)}^{2}\\ & & +\left(\frac{2n+r}{n}+\frac{a(r)}{n-1}-2\right)\sum_{i=1}^{n-1}{\left(h_{ii}^{0\alpha }\right)}^{2}\\ & & -4\sum_{1\leq i<j\leq n}h_{ii}^{0\alpha }h_{jj}^{0\alpha }+\left(\frac{2n+r}{n}-2\right){\left(h_{nn}^{0\alpha }\right)}^{2}\}\\ & \geq & \sum_{\alpha =n+1}^{2m}\left[\frac{r(n-1)+a(r)n}{n(n-1)}\sum_{i=1}^{n-1}{\left(h_{ii}^{0\alpha }\right)}^{2}+\left(\frac{r}{n}\right){\left(h_{nn}^{0\alpha }\right)}^{2}-4\sum_{1\leq i<j\leq n}h_{ii}^{0\alpha }h_{jj}^{0\alpha }\right]. \end{array}$$

We choose $q_{\alpha }$, a quadratic form defined by $q_{\alpha }:R^{n}\to R$ for any α∈{n+1,…,2m},

$$\begin{array}{ccc} q_{\alpha }\left(h_{11}^{0\alpha },h_{22}^{0\alpha },\ldots ,h_{nn}^{0\alpha }\right) & = & \sum_{i=1}^{n-1}\frac{r(n-1)+a(r)n}{n(n-1)}{\left(h_{ii}^{0\alpha }\right)}^{2}\\ & & +\frac{r}{n}{\left(h_{nn}^{0\alpha }\right)}^{2}-4\sum_{1\leq i<j\leq n}h_{ii}^{0\alpha }h_{jj}^{0\alpha }. \end{array}$$

Our aim is to study the constrained extremum problem

$$\min q_{\alpha }$$

with the condition

$$T:h_{11}^{0\alpha }+h_{22}^{0\alpha }+\ldots +h_{nn}^{0\alpha }=k^{\alpha },$$

where $k^{\alpha }$ is a real constant.

We solve the following system of first order partial derivatives:

$$\left\{\begin{array}{c} \frac{\partial q_{\alpha }}{\partial h_{ii}^{0\alpha }}=2\frac{r(n-1)+a(r)n}{n(n-1)}h_{ii}^{0\alpha }-4\left(\sum_{k=1}^{n}h_{kk}^{0\alpha }-h_{ii}^{0\alpha }\right)=0\\ \frac{\partial q_{\alpha }}{\partial h_{nn}^{0\alpha }}=\frac{2r}{n}h_{nn}^{0\alpha }-4\sum_{k=1}^{n-1}h_{kk}^{0\alpha }=0, \end{array}\right.$$

for every i∈{1,…,n−1}, α∈{n+1,…,2m}.

The above system solutions are

$$h_{ii}^{0\alpha }=\frac{2n(n-1)}{\left(n-1)(2n+r)+na(r\right)}k^{\alpha }$$

$$h_{nn}^{0\alpha }=\frac{2n}{2n+r}k^{\alpha },$$

for any i∈{1,…,n−1}, α∈{n+1,…,2m}.

For p∈ W, let F be a 2-form, $F:T_{p}W\times T_{p}W\to R$ defined by

$$F\left(X_{1},X_{2}\right)=\mathrm{Hess}\left(q_{\alpha }\right)\left(X_{1},X_{2}\right)+\langle h^{{}^{\prime }}\left(X_{1},X_{2}\right),\left(\mathrm{grad}q_{\alpha }\right)\left(p\right)\rangle ,$$

where $h^{\prime }$ is the second fundamental form of *W* in $R^{n+1}$, and 〈·,·〉 is the standard inner product on $R^{n}$.

The Hessian matrix of $q_{\alpha }$ is as follows:

$$\mathrm{Hess}\left(q_{\alpha }\right)=\left(\begin{array}{ccccc} \lambda & -4 & \cdots & -4 & -4\\ -4 & \lambda & \cdots & -4 & -4\\ \vdots & \vdots & \ddots & \vdots & \vdots \\ -4 & -4 & \cdots & \lambda & -4\\ -4 & -4 & \cdots & -4 & \frac{2r}{n} \end{array}\right),$$

where $\lambda =2\frac{\left(n-1)(r+2n)+na(r\right)}{n(n-1)}$ is a real constant.

The hyperplane *W* is totally geodesic in $R^{n}$, so we have $\sum_{i=1}^{n}U_{i}=0$, for a vector field $X_{1}\in T_{p}W$. We obtain

$$\begin{array}{cc} F(X_{1},X_{1}) & =\lambda \sum_{i=1}^{n-1}U_{i}^{2}+\frac{2r}{n}U_{n}^{2}-8\sum_{i,j=1(i\neq j)}^{n}U_{i}U_{j}\\ \; & =\lambda \sum_{i=1}^{n-1}U_{i}^{2}+\frac{2r}{n}U_{n}^{2}+4{\left(\sum_{i=1}^{n}U_{i}\right)}^{2}-8\sum_{i,j=1(i\neq j)}^{n}U_{i}U_{j}\\ \; & =\lambda \sum_{i=1}^{n-1}U_{i}^{2}+\frac{2r}{n}U_{n}^{2}+4\sum_{i=1}^{n}U_{i}^{2}\\ \; & \geq 0. \end{array}$$

From Remark 1, the critical point $\left(h_{11}^{0\alpha },\ldots ,h_{nn}^{0\alpha }\right)$ of $q_{\alpha }$ is the global minimum point of the problem. We then achieve Q≥0.

We have thus proved the inequalities (15) and (16), considering infimum and supremum, respectively, over all tangent hyperplanes *V* of $T_{x}N$.

Similarly, we investigated the equality cases of the inequalities (15) and (16). The critical points of Q denoted by

$$h^{c}=\left(h_{11}^{0\;n+1},h_{12}^{0\;n+1},\ldots ,h_{n\;n}^{0\;n+1},\ldots ,h_{11}^{0\;2m},\ldots ,h_{n\;n}^{0\;2m}\right)$$

are the solutions of the following system of linear homogeneous equations:

$$\left\{\begin{array}{c} \frac{\partial Q}{\partial h_{ii}^{0\alpha }}=2\left[\frac{2n+r}{n}+\frac{a(r)}{n-1}-2\right]h_{ii}^{0\alpha }-4\sum_{k\neq i,k=1}^{n}h_{kk}^{0\alpha }=0,\\ \frac{\partial Q}{\partial h_{nn}^{0\alpha }}=2\frac{r}{n}h_{nn}^{0\alpha }-4\sum_{k=1}^{n-1}h_{kk}^{0\alpha }=0,\\ \frac{\partial Q}{\partial h_{ij}^{0\alpha }}=4\left[\frac{2n+r}{n}+\frac{a(r)}{n-1}\right]h_{ij}^{0\alpha }=0,\;\;i\neq j,\\ \frac{\partial Q}{\partial h_{in}^{0\alpha }}=4\left[\frac{2n+r}{n}+\frac{a(r)}{n-1}\right]h_{in}^{0\alpha }=0. \end{array}\right.$$

We find that the critical points satisfy $h_{ij}^{0\alpha }=0$, with i,j∈{1,…,n} and α∈{n+1,…,2m}. However, because of the conditions Q≥0 and $Q(h^{c})=0$, the critical point $h^{c}$ is a minimum point of Q. Thus, both inequalities (15) and (16) satisfy the equality cases if and only if $h_{ij}^{\alpha }=-h_{ij}^{*\alpha },$ for i,j∈{1,…,n}, α∈{n+1,…,2m}. □

**Remark** **2.** *The equality cases of inequalities *(15)* and *(16)* hold for all unit tangent vectors at x if and only if x is a totally geodesic point with respect to the Levi–Civita connection. It is argued that the submanifold N is totally geodesic with respect to the Levi–Civita connection ${\bar{\nabla }}^{0}$ in Equation *(17)*.*

Next, we consider the normalized δ-Casorati curvatures $\delta_{C}\left(n-1\right)$ and $\delta_{C}^{*}\left(n-1\right)$, respectively ${\hat{\delta }}_{C}\left(n-1\right)$ and ${\hat{\delta }}_{C}^{*}\left(n-1\right)$. We then see the following consequences of Theorem (2).

**Corollary** **1.** *Let N be an n-dimensional totally real spacelike submanifold of a 2m-dimensional statistical manifold of a type para-Kähler space form ($\bar{M},\bar{\nabla },P,\bar{g}$). The following δ-Casorati curvatures then satisfy*

*(i)* 
(23) $$\begin{array}{ccc} \rho & \leq & \delta_{C}^{0}\left(n-1\right)+\frac{1}{n-1}C^{0}-\frac{2n}{n-1}{∥H^{0}∥}^{2}\\ & + & \frac{n}{n-1}\bar{g}\left(H,H^{*}\right)+\frac{c}{4}n\left(n-1\right), \end{array}$$
  where $2\delta_{C}^{0}\left(n-1\right)=\delta_{C}\left(n-1\right)+\delta_{C}^{*}\left(n-1\right)$ and $2C^{0}=C+C^{*}$, and
*(ii)* 
(24) $$\begin{array}{ccc} \rho & \leq & {\hat{\delta }}_{C}^{0}\left(n-1\right)+\frac{1}{n-1}C^{0}-\frac{2n}{n-1}{∥H^{0}∥}^{2}\\ & + & \frac{n}{n-1}\tilde{g}\left(H,H^{*}\right)+\frac{c}{4}n\left(n-1\right), \end{array}$$
  where $2{\hat{\delta }}_{C}^{0}\left(n-1\right)={\hat{\delta }}_{C}\left(n-1\right)+{\hat{\delta }}_{C}^{*}\left(n-1\right)$.

Moreover, the equality cases of *(23)* and *(24)* hold identically at all points if and only if h and $h^{*}$ satisfy the condition *(17)*, which implies that N is a totally geodesic submanifold with respect to the Levi–Civita connection.

**Corollary** **2.** *Let N be a spacelike Lagrangian submanifold of the para-Kähler space form ${\bar{M}}_{n}^{2n}$. We then have*

*(i)* 
(25) $$\begin{array}{ccc} \delta_{C}\left(r;n-1\right) & \geq & 2\tau +n^{2}{∥H∥}^{2}-nC+\frac{c}{4}n\left(1-n\right), \end{array}$$
  *where r∈R such that 0<r<n(n−1), and*
*(ii)* 
(26) $$\begin{array}{ccc} {\hat{\delta }}_{C}\left(r;n-1\right) & \geq & 2\tau +n^{2}{∥H∥}^{2}-nC+\frac{c}{4}n\left(1-n\right), \end{array}$$
  *where r∈R such that r>n(n−1).*

*Furthermore, the equality sign of *(25)* and *(26)* holds identically at all points x∈N if and only if N is a totally geodesic submanifold.*

**Remark** **3.** *In a future article, we will investigate the optimality of the inequalities in the previous corollary. Due to additional properties that the second fundamental form h has in the Lagrangian case (see [33], Lemma 3.2), it is expected that the inequalities can be improved, and the case of equality can be achieved for another class of submanifolds.*

**Remark** **4.** *In the main inequalities demonstrated in this section, we established several elementary relationships between some fundamental extrinsic and intrinsic curvature invariants of totally real spacelike submanifolds in statistical manifolds of the type para-Kähler space form. The geometric significance of these inequalities is as follows. It is known that curvature invariants play basic roles in Riemannian geometry and in mathematical physics. It is worth mentioning that, due to the fact that Riemannian invariants model the behavior of a Riemannian space, these invariants are called Riemannian DNA [7]. The extrinsic curvature invariant that measures the shape of submanifolds in the most natural, best agreement with our intuitive, common-sense idea or feeling of curvature very likely is the Casorati curvature [40]. Obtaining elementary relationships between extrinsic and intrinsic curvature invariants is a fundamental problem in modern Riemannian geometry, since it is essential to be able to control the extrinsic quantities relative to intrinsic ones [41]. As inequalities provide such elementary relationships, the relevance of the results stated in this paper is clear. By carefully analyzing the nature of the terms in the main inequalities proved above, one can deduce that the simplest intrinsic curvature invariant, namely, the (normalized) scalar curvature, has an upper bound expressed in terms of some basic extrinsic curvature invariants.*

## 4. Examples

**Example** **1.** *We notice that any para-Kähler manifold is a para-Kähler-like statistical manifold, while para-Kähler space forms provide particular examples of statistical manifolds of type para-Kähler space forms. The prototype of flat para-Kähler spaces is given by the para-Kähler n-plane $E_{n}^{2n}$ (see [42]). The complete classification of para-Kähler space forms of nonzero para-sectional curvature was realized by Gadea and Montesinos-Amilibia [43]. In particular, any totally geodesic totally real spacelike submanifold in such spaces provides examples of submanifolds satisfying the equality case in the inequalities stated above at all points. In the case of non-totally geodesic points, it is clear that the inequalities are strict. Both totally geodesic and non-totally geodesic examples of spacelike Lagrangian submanifolds in $E_{n}^{2n}$ can be found in [32,33,44]. Next, we will present two such examples of submanifolds in $E_{n}^{2n}$.*

**Example** **2.** *The spacelike n-dimensional plane provides a very natural example of a totally geodesic spacelike Lagrangian submanifold of $E_{n}^{2n}$. This submanifold satisfies the equality cases of the inequalities stated above at all points.*

**Example** **3.** *If $\left(a_{1}\left(t\right),a_{n+1}\left(t\right)\right)$ is a non-degenerate spacelike curve in $E_{1}^{2}$, then the submanifold of $E_{n}^{2n}$ defined by*

$$f\left(t,x_{2},\ldots ,x_{n}\right)=\left(a_{1}\left(t\right),0,\ldots ,0,a_{n+1}\left(t\right),x_{2},\ldots ,x_{n}\right),$$

*is a spacelike Lagrangian submanifold without totally geodesic points. In this case, the inequalities stated above are strictly satisfied at all points, and the case of equality cannot be reached.*

**Example** **4.** *On $R^{2n}$ with the coordinates $\left(x_{1},\ldots ,x_{n},y_{1},\ldots ,y_{n}\right)$, we consider the flat affine connection *∇* and the pseudo-Riemannian metric g expressed by*

$$g=\sum_{i=1}^{n}\left(-\alpha dx_{i}^{2}+dy_{i}^{2}\right),$$

*where α≠0 is a real constant. Consider on $R^{2n}$ the almost product structure P given by*

$$P\left(\partial_{x_{i}}\right)=\partial_{y_{i}},\;P\left(\partial_{y_{i}}\right)=\partial_{x_{i}},\;i=1,\ldots ,n.$$

It is then easy to check that $\left(R^{2n},\nabla ,P,g\right)$ is a statistical manifold of the type para-Kähler space form, being a flat para-Kähler-like statistical manifold. Note that the conjugate connection $\nabla^{*}$ is also flat, and $P^{*}$ is given by

$$P^{*}\left(\partial_{x_{i}}\right)=\alpha \partial_{y_{i}},\;P^{*}\left(\partial_{y_{i}}\right)=\frac{1}{\alpha }\partial_{x_{i}}.$$

*Let X be an open set of $R^{n}$, and define an isometric immersion $u:X\to R^{2n}$ by*

$$u\left(y_{1},\ldots ,y_{n}\right)=\left(0,\ldots ,0,y_{1},\ldots ,y_{n}\right).$$

u then defines a spacelike Lagrangian submanifold of $\left(R^{2n},\nabla ,P,g\right)$. Moreover, we have $h=-h^{*}$ and the equality case holds in the inequalities stated above at all points.

## 5. Conclusions

In this paper, we proved new inequalities between extrinsic and intrinsic invariants (δ-Casorati curvatures and a scalar curvature) of totally real spacelike submanifolds in statistical manifolds of the type para-Kähler space form. Furthermore, we investigated the equality cases and obtained some examples. This research may be a challenge for new developments focused on new relationships in terms of various invariants, for different types of statistical submanifolds in this ambient space.

## Data Availability

Not applicable.
